# A Treatise on Neuralgia

**Published:** 1890-08

**Authors:** 


A TREATISE ON NEURALGIA. By E. P. HURD, M. D., Physicians Leisure Library. Series IV., 1890. George S. Davis, Detroit, Mich. Price, in paper, 25 cents; in cloth, 50 cents.

In a neat little volume of 153 pages is considered briefly but ably a subject, the study of which has engaged the attention of writers from time immemorial. The author divides his treatise into ten chapters, treating his subject systematically, and although not being a subject amenable to much original investigation, yet the author cites many cases and experiences that have come under his own observation. The treatment of neuralgia is the important point connected with the whole subject, and the one which surmounts all others. The writer lays much stress upon the constitutional treatment, and herein his little work is to be commended. The local treatment is also ably discussed, together with a general review of all anti-neuralgic remedies and agents up to the present time.

The typography is up to the high standard of this house.

